# *Lactobacillus acidophilus* CRL 1014 improved “gut health” in the SHIME^®^ reactor

**DOI:** 10.1186/1471-230X-13-100

**Published:** 2013-06-11

**Authors:** Katia Sivieri, Martha L Villarreal Morales, Maria A Tallarico Adorno, Isabel Kimiko Sakamoto, Susana M Isay Saad, Elizeu A Rossi

**Affiliations:** 1 Department of Food & Nutrition, Faculty of Pharmaceutical Sciences, São Paulo State University, Araraquara Jau Highway, Km1, Araraquara, SP, Brazil; 2 Department of Hydraulic and Sanitation, University of São Paulo, São Carlos, SP, Brazil; 3 University of São Paulo, Faculty of Pharmaceutical Sciences, 580, Prof. Lineu Prestes Av, São Paulo, SP 05508-000, Brazil

**Keywords:** Gut microbiota, Probiotics, Lactic acid bacteria, Gastrointestinal resource management

## Abstract

**Background:**

How to maintain “gut health” is a goal for scientists throughout the world. Therefore, microbiota management models for testing probiotics, prebiotics, and synbiotics have been developed.

**Methods:**

The SHIME^®^ model was used to study the effect of *Lactobacillus acidophilus* 1014 on the fermentation pattern of the colon microbiota. Initially, an inoculum prepared from human feces was introduced into the reactor vessels and stabilized over 2-wk using a culture medium. This stabilization period was followed by a 2-wk control period during which the microbiota was monitored. The microbiota was then subjected to a 4-wk treatment period by adding 5 mL of sterile peptone water with *L. acidophilus* CRL1014 at the concentration of 10^8^ CFU/mL to vessel one (the stomach compartment). Plate counts, Denaturing Gradient Gel Electrophoresis (DGGE), short-chain fatty acid (SCFA) and ammonium analyses were carried out for monitoring of the microbial community from the colon compartments.

**Results:**

A significant increase (p < 0.01) in the *Lactobacillus* spp. and *Bifidobacterium* spp. populations was observed during the treatment period. The DGGE obtained showed changes in the lactobacilli community from the colon compartments of the SHIME^®^ reactor. The (SCFA) concentration increased (p < 0.01) during the treatment period, due mainly to significant increased levels of acetic, butyric, and propionic acids. However, ammonium concentrations decreased during the same period (p < 0.01).

**Conclusions:**

This study showed the beneficial influence of *L. acidophilus* CRL 1014 on microbial metabolism and lactobacilli community composition for improving human health.

## Background

“Gut health” is a term increasingly used in the scientific literature and by the food industry. It covers multiple positive aspects of the gastrointestinal (GI) tract, such as the effective digestion and absorption of food, the absence of GI illness, normal and stable gut microbiota, effective immune status and a state of well-being [1].

Gut microbiota is composed of different bacterial species, which are involved in the metabolism of nutrients, the maturation of the intestinal epithelium, vasculature and lymphoid tissue, and protection against pathogens [2]. The composition of the intestinal microbiota varies along the gastrointestinal tract in its different compartments [3] and also within specific compartments. For instance, the mucosa seems to harbor a different microbiota than the lumen and very few microorganisms are in direct contact with the epithelium [4]. The majority of bacteria in the adult gut are non-sporing anaerobes, the most numerous include *Bacteroides* spp., *Bifidobacterium* spp., *Eubacterium* spp., *Clostridium* spp., *Fusobacterium* spp., and various gram-positive cocci. Bacteria that are present in lower numbers include *Enterococcus* spp., *Enterobacteriaceae*, methanogens, and dissimilatory sulfate-reducing bacteria [5,6].

Currently, how to maintain intestinal health is a major challenge in medicine. There are many strategies to improve gut health, such as the consumption of a balanced diet that includes large quantities of vegetables [7] and moderate consumption of red meat [8]. Other options involve using the intestinal microbiome or GI barrier modulators, such as probiotics or prebiotics [9]. Indeed, it has been shown that chronic bowel diseases, such as IBD, are associated with adherence of commensal bacteria to the otherwise sterile intestinal epithelium [10]. Selected probiotics might prevent the adhesion of pathogenic bacteria to the intestinal mucosa [11] or restore leaky gut by improving the molecular composition of tight junctions [12].

Some strains of *Bifidobacterium* and *Lactobacillus* have been associated with improved health, resulting in the emergence of probiotics science, the delivery of specific bacteria to the colon or the administration of dietary components that promote the growth of specific bacteria with defined metabolic functions [13].

The Simulator of the Human Intestinal Microbial Ecosystem (SHIME^®^) is an *in vitro* system proven to be a very useful model for nutrition studies, in terms of analysis of the intestinal microbial community composition [14-16]. This study aimed to evaluated the interactions of *Lactobacillus acidophilus* CRL1014 with native microbiota after passing through simulated stomach and small intestine conditions. Finally, the capacity to temporarily modulate the intestinal microbiota after oral administration was investigated using SHIME^®^ reactor.

## Methods

### Preparation of *L. acidophilus* CRL 1014 cells

At weekly intervals, a pure culture of *L. acidophilus* CRL 1014 (CERELA, San Miguel de Tucumán, Argentina) was inoculated into De Man, Rogosa and Sharpe (MRS) broth (Acumedia, Baltimore, USA). Cultures were harvested during the exponential growth, after, they were centrifuged (4000 × g, 10 min, 4°C) and washed with sterile peptone water. The *L. acidophilus* CRL1014 cells were kept at the concentration of 10^8^ CFU/mL in sterile peptone water until use [17].

### Long-term SHIME^®^ run

The SHIME^®^ is a simulator of the human intestinal microbial ecosystem [18,19] in which environmental conditions (pH, residence time, inoculum, and temperature) are controlled to resemble those found in vivo. A SHIME^®^ system consists of five double-jacketed vessels, simulating the stomach, the small intestine, and the ascending, transverse and descending colon, with a total retention time of 72 h (Additional file 1: Figure S1). The reactor setup and the composition of the liquid feed (Table 1), which entered the system three times per day, were previously described by Possemiers et al. [14].

**Table 1:** Ingredients (g) employed for each liter of the basal feed used in the Shime reactor

**Ingredient**	**Quantity necessary for 1 L**
Arabinogalactan (Sigma, USA)	1.0
Pectin (Sigma, USA)	2.0
Xylan (Sigma, USA)	1.0
Potato starch (Unilever, Brazil)	3.0
Glucose (Sigma, USA)	0.4
Yeast extract (Sigma, USA)	3.0
Peptone (Sigma, USA)	1.0
Mucin (Sigma, USA)	4.0
Cystein (Sigma, USA)	0.5
Sterile distilled water	qsp

The three colon vessels of the SHIME^®^ reactor were inoculated with bacteria from a fecal sample of a healthy 22-year-old adult female with no history of antibiotic treatment 6 months prior to the study. Aliquots (10 g) of fresh fecal samples were diluted and homogenized with 100 mL of sterilized phosphate buffer (0.1 mol/L, pH 7), containing 1 g/L sodium thioglycolate as the reducing agent.

The microbial inoculum was stabilized over a period of 2-wk on a carbohydrate-based medium and allowed to adapt to the specific environmental conditions of the ascending, transverse and descending colon, in terms of pH range, retention time and available carbon sources [14,15]. Upon stabilization, the SHIME^®^ run included 2- wk of basal period (to quantify all steady-state bacterial parameters which were used as starting point to evaluate the effect of a specific treatment), and a 4-wk of treatment period, in which 5 mL of 10^8^ CFU/mL of *L. acidophilus* CRL 1014 were added once per day to the stomach compartment. Finally, a 2-week washout period without the addition of *L. acidophilus* CRL 1014 was observed.

### Microbiological analysis

At weekly intervals, throughout the entire experimental period, (basal, treatment and washout), 5 mL samples were collected from the reactors for microbiological examinations. The analysis of the intestinal microbiota composition was based on the enumeration of total aerobic and anaerobic bacteria, *Enterococcus* spp., *Lactobacillus* spp., *Bifidobacterium* spp., enterobacteria, and *Clostridium* spp. One mL of a sample taken from each reactor was suspended in 99 mL of peptone water. Serial dilutions were prepared and inoculated into selective culture media, as follows: total aerobic and anaerobic counts: Standard Methods agar (Acumedia, Baltimore, USA; 37°C/48 h); *Enterococcus* spp.: KF *Streptococcus* agar (Acumedia, Baltimore, USA; 37°C/48 h) [16]; *Lactobacillus* spp.: MRS agar (Merck, Germany; 37°C/48 h, under anaerobiosis). For *Bifidobacterium* spp. counts was used the Bifidobacterium formulated medium BIM-25 (Difco, France; 37°C/72 h, Anaerobic System, Probac, Brazil) according Munoa & Pares [20], Enterobacteria: MacConkey agar (Acumedia, Baltimore, USA; 37°C/48 h) and *Clostridium* spp.: RCA Agar (Difco, France; 37°C/48 h, Anaerobic System, Probac, Brazil) [21].

### Analysis of short-chain fatty acids and ammonium

Once a week, throughout the entire experimental period (basal, treatment and washout), samples were collected from the reactors for analysis SCFA and ammonium. The analysis was carried out in triplicates.

Every week, the levels of short-chain fatty acids (SCFA) were determined from samples collected from the reactors and frozen to -20°C. The SCFA were extracted with diethyl ether and determined using a gas chromatograph equipped with a flame-ionization gas detector, a capillary split/splitless injector and an HP-INNOWAX column with a 30 m × 0.25 mm × 0.25 μm inlet (Shimadzu GC2010), using hydrogen as the carrier gas at a flow rate of 1.56 mL/min. The temperatures of the column, injector and detector were 170, 250 and 280°C, respectively [22].

The ammonia content was determined using a selective ion meter (710A model, Orion) coupled to an ammonia selective-ion electrode (Orion 95–12). The apparatus was calibrated using 0.1 M standard ammonium chloride solutions, at the concentrations of 10, 100, and 1000 mg/L of ammonia. To every 25 mL of sample, 0.5 mL of ISA solution (Ionic Strength Adjuster, Orion – a pH-adjusting and ionic force solution) was added. All measurements were carried out at 25°C [23].

### Composition of the *Lactobacillus* community

DNA was extracted from 2 mL of sample using the QIAamp DNA Stool Mini Kit (Qiagen, Hilden, Germany), according to the manufacturer’s protocol. DNA yield was quantified using a NanoDrop ND-1000 spectrophotometer (NanoDrop Technologies, Willmington, USA).

### DGGE analysis

The diversity of the *Lactobacillus* community in samples taken throughout model operation was assessed by DGGE. To prevent a low amplicon yield a nested PCR approach was used as previously described [24]. This involved a first round of PCR with primers Bact27f (5′-GTTTGATCCTGGCTCAG-3′) [25] and 1492R (5′- CGG CTA CCT TGT TAC GAC-3′) [26], followed by a second PCR with primers Lab159f (5′-GGAAACAGATGCTAATACCG-3′) and Lab677-GCr (5′-GCCCGGGGCGCGCCCCGGGCGGGGCGGGGGCACGGGGGGGCACCGCTACACATGGAG-3′) [24].

PCR was performed using the GoTaq^®^ Green Master Mix kit (Promega, USA). Samples were amplified in a Veriti^®^ 96-Well Thermal Cycler (Applied Biosystems, USA) by using the following program: initial denaturation at 94°C for 2 min; 35 cycles of denaturation at 94°C for 30 s, annealing at primer-specific temperature for 40 s, elongation at 72°C for primer-specific time, and extension at 72°C for 5 min, followed by a final cooling to 4°C. The annealing temperature and elongation time was set at 52°C/1.30 min with primers Bact27f and 1492r and 60°C/1 min with primers Lab159f and Lab677-GCr. The PCR products that were used as templates in nested PCR were purified with the QIAquick PCR Purification Kit (Qiagen, USA).

DGGE analysis of PCR amplicons was based on the protocol described by Muyzer et al. [27] by using the DCode System (Bio-Rad Laboratories, Hercμles, CA, USA). Electrophoresis was done as described previously [24] in an 8% polyacrylamide gel with a denaturant gradient of 30–50% (100% was defined as 40% formamide and 7 M urea) for 16 h at 85 V in a 0.5× TAE buffer at a constant temperature of 60°C. Gels were stained with silver nitrate by the method of Sanguinetti et al. [28], scanned at 400 d.p.i., and further analyzed by the BioNumerics 6.0 software (Applied Maths). The distance matrices of each DGGE based on the Pearson correlation similarity coefficient to cluster the samples was analyzed using the BioNumerics software (Applied Maths).

Bands of interest in the lactobacilli community fingerprint were excised from the gel and transferred into 25 μl of TE buffer, and incubated overnight at 37°C to allow diffusion of the DNA. Two microliters of the eluted DNA were used for reamplification with the GC-clamped primer by using the conditions described above and the PCR products generated were checked by DGGE. Only PCR products which yielded a single band and comigrated with the original band were purified by the QIAprep spin miniprep kit (Qiagen, USA) and were subjected to DNA sequence analysis at the genomic facilities of The Human Genome Research Center (HGRC), at the University of São Paulo. BLAST searches were performed to determine the closest known relatives of the partial rRNA gene sequence obtained in GenBank.

### Nucleotide sequence accession numbers

The sequences of the 16S rDNA fragments were deposited in the GenBank database. The accession numbers of the 4 sequences are as follows (band code in parentheses): KF054352 (lac1), KF054351 (lac2), KF054353 (lac3) and KF054350 (lac4).

### Statistical analysis

The significance of all results was investigated with one-way ANOVA, and individual means were compared using the Tukey’s test (p < 0.01), using the statistical software Sigma Stat 5.0.

## Results

### Plate count data

Table 2 shows the microbial counts obtained for the flasks that simulated the ascendant, transverse and descendant colon of the SHIME^®^ reactor. Using traditional selective growth media, the microbiological analyses revealed that the administration of *L. acidophilus* CRL1014 influenced the composition of the intestinal microbial community.

**Table 2:** **Average plate count measurements (±SEM), expressed in log CFU mL**^**1**^**, for the different microbial groups, SHIME compartments and periods**

	**Basal**		**Treatment**			**Washout**		
	^**1st **^**week**	^**2nd **^**week**	^**1st **^**week**	^**2nd**^**week**	^**3th **^**week**	^**4th **^**week**	^**1st**^**week**	^**2nd**^**week**
**Ascending colon**								
*Enterococcus* spp	5.20^a^ ± 0.01	5.40^a^ ± 0.02	6.30^b^ ± 0.10	7.25^c^ ± 0.04	7.20^c^ ± 0.06	8.05^d^ ± 0.21	7.00^c^ ± 0.02	7.00^c^ ±0.01
enterobacteria	7.35^d^ ± 0.41	6.00^c^ ± 0.30	6.20^c^ ± 0.32	6.40^c^ ± 0.01	6.72^c^ ± 0.22	6.80^c^ ± 0.32	5.68^b^ ± 0.01	4.20^a^ ± 0.05
*Lactobacillus* spp	5.17^a^ ± 0.30	5.23^a^ ± 0.22	7.78^b^ ± 0.03	7.77^b^ ± 0.01	8.28^c^ ± 0.05	7.88^bc^ ± 0.01	7.20^b^ ± 0.04	5.40^a^ ± 0.04
*Bifidobacterium* spp	5.80^a^ ± 0.22	6.90^b^ ± 0.55	7.77^c^ ± 0.01	7.86^c^ ± 0.05	7.88^c^ ± 0.06	7.97^c^ ± 0.06	7.68^c^ ± 0.03	7.61^c^ ± 0.04
*Clostridium* spp	7.68^a^ ± 0.02	8.32^b^ ± 0.02	8.69^b^ ± 0.01	9.39^bc^ ± 0.01	12.39^d^ ± 0.01	12.30^d^ ± 0.02	12.40^d^ ± 0.01	12.39^d^ ± 0.01
Total aerobes	7.14^a^ ± 0.41	7.84^a^ ± 0.05	7.72^a^ ± 0.01	7.42^a^ ± 0.02	7.55^a^ ± 0.04	7.32^a^ ± 0.01	7.30^a^ ± 0.01	7.42^a^ ± 0.05
Facultative anaerobes	7.66^a^ ± 0.03	8.20^a^ ± 0.02	7.80^a^ ± 0.02	7.60^a^ ± 0.04	7.40^a^ ± 0.01	7.30^a^ ± 0.03	7.46^a^ ± 0.02	7.50^a^ ± 0.01
**Transverse colon**								
*Enterococcus* spp	4.14^a^ ± 0.22	4.44^a^ ± 0.02	7.19^b^ ± 0.01	6.84^b^ ± 0.01	7.45^b^ ± 0.03	7.50^b^ ± 0.05	7.00^b^ ± 0.04	7.00^b^ ± 0.01
enterobacteria	6.23^b^ ± 0.46	5.15^a^ ± 0.15	5.00^a^ ± 0.01	6.30^b^ ± 0.01	6.00^b^ ± 0.01	6.00^b^ ± 0.01	6.84^b^ ± 0.01	6.86^b^ ± 0.02
*Lactobacillus* spp	5.20^a^ ± 0.02	5.19^a^ ± 0.02	7.72^b^ ± 0.02	7.35^b^ ± 0.05	7.24^b^ ± 0.01	7.17^b^ ± 0.05	8.33^b^ ± 0.05	8.40^b^ ± 0.01
*Bifidobacterium* spp	5.80^a^ ± 0.07	5.00^a^ ± 0.01	7.18^b^ ± 0.14	7.86^b^ ± 0.05	7.43^b^ ± 0.01	7.31^b^ ± 0.14	7.79^b^ ± 0.05	7.80^b^ ± 0.02
*Clostridium* spp	6.61^a^ ± 0.01	6.62^a^ ± 0.01	7.27^a^ ± 0.01	8.39^b^ ± 0.01	8.26^b^ ± 0.03	8.53^b^ ± 0.23	10.39^c^ ± 0.01	10.40^c^ ± 0.01
Total aerobes	7.00^a^ ± 0.02	7.20^a^ ± 0.20	7.74^a^ ± 0.02	7.32^a^ ± 0.11	7.82^a^ ± 0.02	7.86^a^ ± 0.03	8.69^b^ ± 0.22	8.64^b^ ± 0.10
Facultative anaerobes	6.32^a^ ± 0.07	6.30^a^ ± 0.01	6.00^a^ ± 0.41	6.20^a^ ± 0.13	6.10^a^ ± 0.22	6.30^a^ ± 0.01	7.80^ab^ ± 0.01	8.10^b^ ± 0.11
**Descending colon**								
*Enterococcus* spp	4.30^a^ ± 0.02	4.32^a^ ± 0.01	7.08^b^ ± 0.01	6.66^b^ ± 0.02	6.75^b^ ± 0.05	6.76^b^ ± 0.04	7.08^b^ ± 0.02	7.06^b^ ± 0.01
enterobacteria.	7.46^b^ ± 0.06	6.00^a^ ± 0.01	6.15^a^ ± 0.15	6.30^a^ ± 0.01	6.00^a^ ± 0.01	6.00^a^ ± 0.01	5.67^ab^ ± 0.01	5.70^ab^ ± 0.01
*Lactobacillus* spp	5.36^a^ ± 0.01	5.38^a^ ± 0.02	7.46^b^ ± 0.02	7.23^b^ ± 0.06	7.13^b^ ± 0.09	6.53^b^ ± 0.53	7.20^b^ ± 0.01	7.27^b^ ± 0.08
*Bifidobacterium* spp	6.79^b^ ± 0.04	5.72^a^ ± 0.12	7.15^c^ ± 0.01	7.53^c^ ± 0.01	6.84^bc^ ± 0.15	7.37^c^ ± 0.03	7.55^c^ ± 0.01	7.47^c^ ± 0.01
*Clostridium* spp	7.72^a^ ± 0.01	7.74^a^ ± 0.06	8.48^b^ ± 0.01	9.39^c^ ± 0.01	10.50^d^ ± 0.15	10.50^d^ ± 0.02	10.34^d^ ±0.02	10.38^d^ ± 0.44
Total aerobes	7.24^a^ ± 0.07	7.53^a^ ± 0.01	7.44^a^ ± 0.01	7.47^a^ ± 0.42	7.58^a^ ± 0.09	7.86^a^ ± 0.05	8.00^a^ ± 0.01	8.20^a^ ± 0.45
Facultative anaerobes	7.39^b^ ± 0.11	6.30^a^ ± 0.01	6.59^a^ ± 0.01	6.39^a^ ± 0.02	6.40^a^ ± 0.01	6.00^a^ ± 0.03	7.69^b^ ± 0.01	7.63^b^ ± 0.05

Different letters indicate significantly different results (p < 0.01) in same microbial group and same compartment (ascending, transverse, and descending colon), during basal, treatment and washout period.

Plate counts were used to assess the capacity of *L. acidophilus* CRL1014 to temporarily colonize the colon during a simulated long term administration and to investigate the effects on the composition of the indigenous microbial community in the SHIME^®^. As reflected in the plate count data (Table 2), the administration of *L. acidophilus* to the system induced a significant increase (p < 0.01) in lactobacilli and bifidobacteria counts, with a concentration increase of at least 2 log CFU in all colon compartments. However, in all colon compartments, a high increase in clostridia starting from the second week of the basal period in ascending colon and first week of the treatment in transverse and descending colon was observed (Table 2).

For other microbial groups, such as total aerobes, facultative anaerobes, and enterobacteria, there were no significant changes in the populations of these microorganisms during the treatment period.

### Effects of the long-term treatment on the *Lactobacillus* community structure

**Figure 1:**
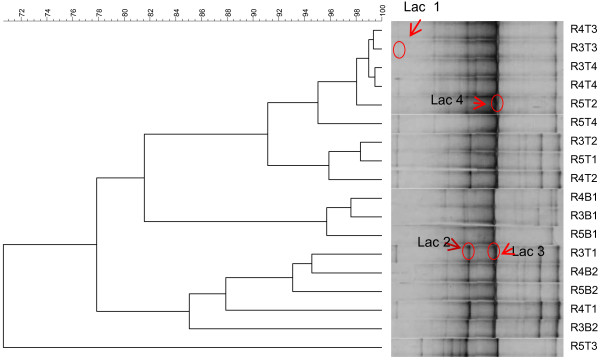
**UPGMA dendrogram illustrating the correlation between the different denaturing gradient gel electrophoresis (DGGE) profiles from lactobacillus community obtained from the samples of the SHIME^®^ compartments supplemented with the *L. acidophilus *CRL 1014 strain throughout model operation.** Sequence characterization of the excised fragments indicated the presence of lac 1, *L. acidophilus;* lac 2, *Lactobacillus casei*; lac 3, *Lactobacillus johnsonii* and Lac 4, *Lactobacillus sakei.* R3: ascending colon, R4: transverse colon, R5: descending colon. B1, and B1: basal period, T1, T2, T3, and T4: treatment period with *L. acidophilus* 1014.

DGGE analysis was used to monitor qualitative changes in the composition and structure of the *Lactobacillus* communities in the three compartments simulating the colon conditions (Figure 1).

Clustering of the specific DGGE fingerprints for lactobacilli (Figure 1) indicated that the treatment had effect on the composition of the *Lactobacillus* community, after 4 weeks of treatment with *L. acidophilus* CRL1014, higher similarity values (>90%) were found between reactors 3, 4, and 5 (Figure 1). Finally, after two wk the treatment period was finished and the highest similarity values (99 to 91%) were found in all colon regions between the last week of treatment and the washout period (Figure 1).

Based on the DGGE fingerprint analysis of the colon microbial community, several shifts in bands or changes in band intensity were observed. To identify the bacterial species that were responsible for those changes, DNA fragments from bands of interest were excised from the DGGE gel, isolated, and finally sequenced. We were not able to obtain sequences from all bands but four. The successfully sequenced rDNA fragments were marked as lac 1, lac 2, lac 3, and lac 4. The band marked as lac1 migrates to the same position as the fragment obtained from pure cultures of *L. acidophilus* CRL1014 and showed a 99% identity with *L.acidophilus* (JQ031741.1). The fragments lac2 and lac3 demonstrated high identity (>98%) to *Lactobacillus casei (JQ412731.1) and Lactobacillus johnsonii (*AB186343.1), respectively. Finally, the band marked “lac 4” was dominant during the whole experimental period and had 99% of similarity with *Lactobacillus sakei* (GI,AB609050.1).

### Ammonium concentration and fermentation capacity

Short chain fatty acids (SCFA) analysis and ammonium production are often used to characterize microbial metabolism.

**Figure 2:**
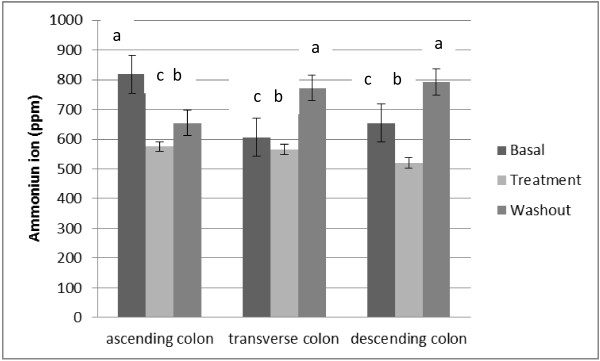
**Average ammonium ion production (ppm) in SHIME^®^ run, during basal, treatment and washout period.** Statistically significant differences among the samples were investigated with one-way ANOVA (samples with the same letter on the top of the bar are not statistically different, *P < 0.05*).

During the treatment period with *L. acidophilus*, the values of concentration of ammonium ion decreased significantly (p < 0.01) in all the regions investigated (Figure 2).

**Figure 3:**
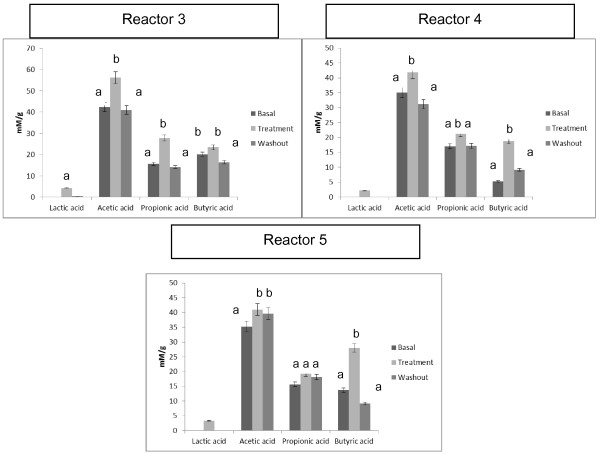
**Metabolic activity (short chain fatty acid (SCFA) acetic acid, propionic acid and butyric acid) and lactic acid of the microbial community in reactor three, four and five from the basal, treatment and washout periods in the SHIME^®^.** Reactor 3: simulates the ascending colon; reactor 4: simulates the transverse colon; reactor 4: simulates the descending colon. Statistically significant differences among the samples were investigated with one-way ANOVA. Samples with the same letter on the top of the bar are not statistically different, (p < 0.01).

Figure 3 depicts the production of acetate, propionic, and butyric acids during the basal, treatment, and washout periods in the SHIME^®^ vessels. During treatment with *L. acidophilus* CRL 1014, a significant increase (p < 0.01) occurred in the production of acetate acid in all the reactors analyzed. However, the greatest concentration of these acids occurred in vessel three, which simulates the ascending region of the colon. The propionic acid showed a significant increase (p < 0.01) in reactors 3 and 4 (ascending and transverse colon) and the butyric acid increased significantly (p < 0.01) in reactors 4 and 5 (transverse and descending colon). In the washout period, the levels of SCFA diminished in all vessels. The highest SCFA production consisted of acetic acid in all vessels (Figure 3). However, in reactor 5 the highest increase was in propionic acid. Lactic acid was only observed for the treatment period in all vessels (ascending, transverse and descending).

## Discussion

The intestinal microbiota is located in difficult-to-access areas of the digestive tract. Therefore, as it requires invasive methods for collection, it is a limiting factor for a more precise analysis [29]. An investigative alternative is the use of continuous or semi-continuous models simulating the large intestine. The continuous models were validated based on the intestinal contents of sudden death victims [30]. The advantages of this model include ease-of-use, the possibility of using radioactive substances and low cost [29].

The appropriate selection of probiotic strains forms the basis for further development of supplements and food products, as well as for planning future clinical trials. *In vitro* studies are useful for evaluating the safety and efficiency of probiotic strains. Recent advances in science have revealed many mechanisms by which probiotics exert health-promoting effects in humans and laboratory animals [6]. In this paper, we evaluated the interactions of *Lactobacillus acidophilus* CRL1014 with the native microbiota, using the Simulator of Human Intestinal Microbial Ecosystem (SHIME^®^).

The results with plate count showed that the *L. acidophilus* CRL1014 strain is able to modulate the native intestinal microbiota. *Lactobacillus* spp. and *Bifidobacteruim* spp. were increased during the treatment period. The population of *Clostridium* spp. was increased 2–3 cycles log during the treatment period in the three regions of the colon evaluated. Bacteria belonging to this genus may be harmful, due to their metabolic activity and the pathogenic character of some species [31]. *Clostridium* species may be involved in inflammatory processes of intestinal diseases [32]. In a previous study with *E. faecium* CRL 183, the same behavior was observed [33]. However, according to Possemiers et al. [34], high clostridia counts are not necessarily associated with negative health effects, as many members of the Clostridia group are associated with the production of health-promoting short chain fatty acids.

Structural analysis of the colon lactobacilli populations using PCR-DGGE showed that administration of *L. acidophilus* CRL 1014 affected the lactobacilli populations. Clustering of the lactobacilli DGGE patterns showed that samples from the treatment period were grouped together. The DGGE and sequencing analysis also showed that the *Lactobacillus acidophilus* effect was not restricted to the ascending colon alone, but was also visible in the distal colon vessels. After the end of the treatment period, it was possible to observe the higher similarity values (97.73%) between treatment and washout periods in the colon ascendant. *Lactobacillus acidophilus* CRL 1014 probably temporarily colonized the simulated gut, while the overall ecological characteristics of the indigenous microbiota were modulated and preserved.

In this study, beneficial effects of *L. acidophilus* CRL 1014 were observed in terms of microbial metabolism. Production of SCFA is considered beneficial to the host, because these compounds protect against pathogens [35], stimulate the immune responses [36], decrease cholesterol synthesis [37], enhance muscular contractions [38], and may protect the colon against cancer development [39].

In all vessels, increased amounts of SFCA (acetate, propionate and butyrate) during the treatment period were observed. The rate and amount of SCFA production depend on the species and number of microorganisms present in the colon, the substrate source, and the gut transit time. The acid with the highest production was acetate. SCFAs are readily absorbed. Acetate, the main SCFA in the colon, is readily absorbed and transported to the liver and, therefore, is less metabolized in the colon. Acetate also enters the peripheral circulation to be metabolized by peripheral tissues [40,41].

Specific SCFA may reduce the risk of developing gastrointestinal disorders, cancer, and cardiovascular disease. Acetate, the main SCFA in the colon, can increase cholesterol synthesis after absorption. However, propionate, a gluconeogenerator, can inhibit cholesterol synthesis. Therefore, substrates that are able to decrease the acetate: propionate ratio may reduce serum lipids and possibly cardiovascular disease risk [42].

The ability to reduce serum cholesterol is a highly desirable attribute of probiotic cultures as a dietary component [43]. Certain strains of *L. acidophilus* have the ability to assimilate cholesterol. This was shown by the appearance of cholesterol in the cells during growth, which was associated with decreases in the concentrations of cholesterol in the growth medium [42]. In a previous study, our researcher group observed the ability of *Lactobacillus acidophilus* CRL 1014 to remove 13% of the cholesterol *in vitro*[43]. Binding of cholesterol with bile acids and inhibition of micelle formation combined with the effect of fermentation on short chain fatty acid (SCFA) production were mechanisms that had been proposed to explain the potential cholesterol-lowering effects of *L. acidophilus* strains [44,45].

A significant increase in butyrate concentration was observed in the vessels simulating the transverse and descendant colon. The same results were observed in recent investigation with *Enterococcus faecium* CRL 183 [33]. Butyrate is considered the preferred fuel of the epithelial cells of the colon, which derive 70% of their energy from the oxidation of this substrate. Butyrate also reduces the expression of proinflammatory cytokines of tumor necrosis factor-α (TNF-α), TNF-β, interleukine-6 (IL-6), and IL-1β through activation of the nuclear growth inhibiting factor kB (NF-kB) [42]. In addition, it has been proposed that butyrate reduces the risk of colon cancer, due to its ability to inhibit the genotoxic capacity of nitrosamines and hydrogen peroxide, as well as to induce different levels of apoptosis, differentiation and cessation of the cellular cycle of colon cancer in animal models [46].

Other important observation in terms of the metabolic activity was the ammonium ion production, which is a marker for proteolytic activity of the microbial population. In the ascending and descending colon, a significant (p < 0.01) reduction (29.8 and 20.5%, respectively) in the ammonium ion production during *L. acidophilus* treatment was observed. Usually, colon cancer occurs in the distal parts, because of higher concentrations of more hazardous compounds due to proteolysis and a higher pH, it is often the goal to extend sugar fermentation towards the distal parts of the colon [47]. During the treatment with *L. acidophilus* decrease proteolytic activity of the microbial population was observed.

## Conclusion

This study indicated the usefulness of *in vitro* methods that simulate the colon region and showed the positive influence of *L. acidophilus* CRL 1014 on microbial metabolism and lactobacilli community composition.

## Competing interests

The authors declare that they have no competing interests.

## Authors’ contributions

KS and EAR designed the experiment and wrote the research article. MAT performed the SFCA experiments. KS and MLVM performed the molecular and data analyses. All authors listed here were involved in drafting the manuscript and they have read and approved the final version for publication.

## Pre-publication history

The pre-publication history for this paper can be accessed here:

http://www.biomedcentral.com/1471-230X/13/100/prepub

## Supplementary Material

Additional file 1: Figure S1 Schematic representation of the Simulator of the Human Intestinal Microbial Ecosystem (SHIME^®^). Possemiers et al. [31]. Vessel 1: stomach; vessel 2: small intestine; vessel 3: ascending colon; vessel 4: transverse colon; vessel 5: descending colon. Click here for file
