# Supplementary material for: Lactobacillus acidophilus CRL 1014 improved “gut health” in the SHIME® reactor
Source: BMC Gastroenterol. 2013 Jun 11;13:100. doi: 10.1186/1471-230X-13-100 (PMC3700768; doi:10.1186/1471-230X-13-100)
Supplement: Additional file 1: Figure S1 — Schematic representation of the Simulator of the Human Intestinal Microbial Ecosystem (SHIME®). Possemiers et al. [31]. Vessel 1: stomach; vessel 2: small intestine; vessel 3: ascending colon; vessel 4: transverse colon; vessel 5: descending colon. [file 1471-230X-13-100-S1.docx]

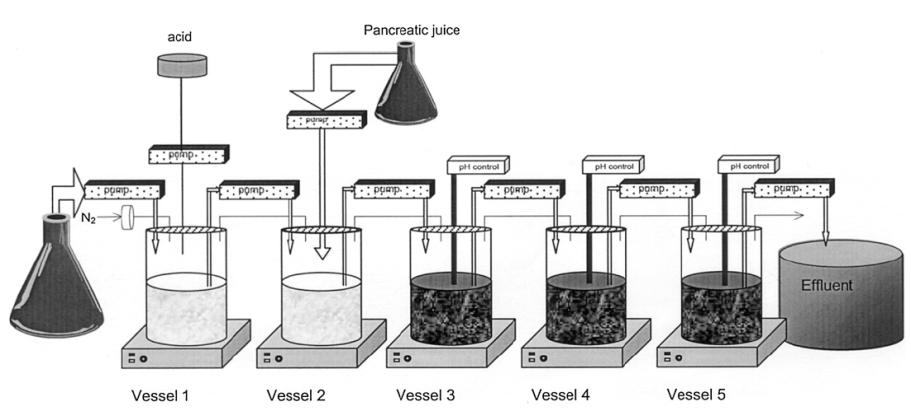


Additional file 1: Figure S1. Schematic representation of the Simulator of the Human Intestinal Microbial Ecosystem (SHIME®). Possemiers et al. [31]. Vessel 1: stomach; vessel 2: small intestine; vessel 3: ascending colon; vessel 4: transverse colon; vessel 5: descending colon.
